# Naturally ornate RNA homo-oligomeric complexes

**DOI:** 10.1063/4.0001088

**Published:** 2025-10-27

**Authors:** Rachael Kretsch, Yuan Wu, Svetlana A Shabalina, Hyunbin Lee, Grace Nye, Eugene V Koonin, Alex Gao, Wah Chiu, Rhiju Das

**Affiliations:** 1 Stanford University, Stanford, CA, USA; 2 National Institutes of Health, Bethesda, MD, USA

## Abstract

Myriad families of natural RNAs have been proposed, but not yet experimentally shown, to form biologically important structures. Cryogenic electron microscopy revealed the three-dimensional structures of three large ornate bacterial RNAs at 2.9-3.1 Å. Without precedent among previously characterized natural RNA molecules, Giant, Ornate, Lake- and Lactobacillales-Derived (GOLLD), Rumen-Originating, Ornate, Large (ROOL), and Ornate Large Extremophilic (OLE) RNAs form homo-oligomeric complexes whose stoichiometries are retained at concentrations lower than expected in the cell. OLE RNA forms a dimeric complex with long co-axial pipes spanning two monomers. Both GOLLD and ROOL form distinct RNA-only multimeric nanocages with diameters larger than the ribosome. Extensive intra- and intermolecular A-minor interactions, kissing loops, an unusual A-A helix, and other interactions stabilize the three complexes. Sequence covariation analysis of these large RNAs reveals evolutionary conservation of intermolecular interactions, supporting the biological importance of large, ornate RNA quaternary structures that can assemble without any involvement of proteins.

